# Sialidosis type I: How to alleviate disabling myoclonic seizures?—A multicenter analysis of eight cases and review of the literature

**DOI:** 10.1002/epi4.70233

**Published:** 2026-02-10

**Authors:** Janina Gburek‐Augustat, I‐Chun Lee, Marica Rubino, Vehap Topçu, Melissa Chavez‐Castillo, Shao Ching Tu, Marwan Shinawi, Isabel Alfradique‐Dunham, Manouela Valtcheva, Astrid Adarmes‐Gómez, Daniel Macias‐Garcia, Laura Laura Muñoz‐Delgado, Silvia Jesús, Pablo Mir, Andreas Merkenschlager, Antonietta Coppola

**Affiliations:** ^1^ Department of Pediatric Neurology University Hospital for Children Leipzig Germany; ^2^ Department of Pediatrics Taichung Tzu chi General Hospital Taichung Taiwan; ^3^ Department of Neuroscience, Reproductive Sciences and Odontostomatology Federico II University of Naples Naples Italy; ^4^ Acibadem Labgen Genetic Diagnosis Center Istanbul Türkiye; ^5^ Pediatric Epilepsy Clinic‐Hospital Civil de Guadalajara Guadalajara Mexico; ^6^ Department of Pediatrics Division of Genetics and Genomic Medicine, Washington, University School of Medicine St. Louis Missouri USA; ^7^ Department of Neurology, Washington University School of Medicine St. Louis Missouri USA; ^8^ Unidad de Trastornos del Movimiento Servicio de Neurología, Instituto de Biomedicina de Sevilla, IBiS/Hospital Universitario Virgen Del Rocío/Universidad de Sevilla Sevilla Spain; ^9^ Centro de Investigación Biomédica en Red Sobre Enfermedades Neurodegenerativas (CIBERNED), Instituto de Salud Carlos III Madrid Spain; ^10^ Departamento de Medicina, Facultad de Medicina Universidad de Sevilla Sevilla Spain; ^11^ Epilepsy Center University Hospital Federico II Naples Italy

**Keywords:** ataxia, lysosomal storage disorder, myoclonic seizures, *NEU1*, sialidosis type 1, ST‐1, ultra‐rare disease

## Abstract

**Objective:**

Sialidosis type I (ST‐1) is an autosomal‐recessive, very rare, progressive lysosomal storage disorder caused by pathogenic variants in *NEU1*. It is clinically characterized by progressive ataxia, myoclonic seizures (MS), bilateral tonic–clonic seizures (BTCS), and distinctive ophthalmological findings. Given the lack of curative options, in this study, we investigated symptomatic treatment strategies, with a particular focus on the efficacy of antiseizure medications (ASMs).

**Methods:**

We describe the clinical course of a patient followed from diagnosis to 18 years of age, and review seven additional cases from our cohort. In parallel, we conducted a narrative review of the literature (PubMed, January 2010–September 2025) to identify published reports containing therapeutic data.

**Results:**

Therapeutic responses were evaluated in a total of 33 cases (8 from our cohort, 25 from published sources). Although available data are insufficient to define standardized treatment guidelines, some ASMs, such as ACZ, PER, LEV, VPA, CZP, and ZNS, demonstrated fairly consistent efficacy in managing MS and BTCS. Sodium oxybate or deep‐brain stimulation may be considered in refractory cases.

**Significance:**

Prospective documentation of clinical course and treatment outcomes—ideally through an international registry—is crucial to improve patient care and inform therapeutic strategies.

**Plain Language Summary:**

Sialidosis type I (ST‐1) is a very rare genetic disorder causing movement problems and seizures, with no cure available yet. We followed 8 patients and reviewed 25 published cases to assess treatments focusing on myoclonic seizure (MS) control. Some antiseizure medications showed benefit. However, we have too little data to make clear recommendations. To improve patients' treatment and to choose the most appropriate therapy, it would be important to follow patients over a longer period of time, for example, in an international registry.

**Key points**

- Sialidosis type I (ST‐1) is a very rare, inherited lysosomal storage disorder caused by *NEU1* gene mutations. It typically manifests in late childhood or adolescence with debilitating myoclonic seizures (MS), bilateral tonic–clonic seizures (BTCS), ataxia, and a cherry‐red macula spot.
- This study longitudinally followed one patient from diagnosis at age 6 to age 18, presented data on seven additional genetically confirmed ST‐1 cases, and reviewed the literature with a focus on treatment responses.
- Some antiseizure medications provided benefit in controlling MS and BTCS, although effects were sometimes transient.
- Data remain too limited to support definitive treatment recommendations.
- International collaboration is urgently needed to establish a registry for systematic collection of clinical and treatment data, as no disease‐modifying therapies currently exist.

## 1 INTRODUCTION

Sialidosis is a very rare, progressive lysosomal storage disorder with an estimated incidence of 1 in 4 000 000 live births in the Caucasian population.^1^ It follows an autosomal recessive inheritance pattern and results from biallelic pathogenic variants in *NEU1* gene, which was first identified in 1996.^2^ To date, over 90 *NEU1* rare variants—predominantly missense—have been described.^3^ Deficiency of α‐N‐acetylneuraminidase (sialidase) leads to abnormal accumulation and urinary excretion of oligosaccharides and glycolipids.

Clinically, sialidosis is divided into two types. Type I (late‐onset, normosomatic) is characterized by residual NEU1 activity of ~1–5%, whereas type II (early‐onset, dysmorphic) exhibits <1% residual activity.^4^ Type II, first recognized by Spranger et al. (1968), presents at birth or in early infancy.^5^ It is subdivided into congenital/neonatal/infantile (0–12 months), and juvenile (13 months–20 years) forms, frequently complicated by hydrops fetalis, ascites, or early death.

In contrast, sialidosis type I (ST‐1)—first described by Durand et al. in 1977—presents in late childhood or adolescence and progresses more slowly.^6^ It is characterized by myoclonic seizures (MS), bilateral tonic–clonic seizures (BTCS), ataxia, and a cherry‐red macular spot. Nevertheless, individuals with ST‐1 endure substantial morbidity, particularly from MS, which compromises motor function and quality of life. MS in ST‐1 is typically stimulus‐sensitive (for example to movement or emotional stress), and neurophysiological findings support a cortical generator. However, due to the precisely synchronous bilateral discharges without interhemispheric time lag, a subcortical mechanism has also been discussed.^7^, ^8^, ^9^

Milder or atypical presentations of ST‐1 are likely underdiagnosed, implying that the disease is more common than reported.^10^, ^11^

Although enzyme replacement, pharmacological chaperones, and gene therapies show theoretical promise, no disease‐modifying treatments are currently on the horizon.^11^, ^12^, ^13^, ^14^ To date, only two natural‐history studies (NCT06316752; NCT00029965) are listed at ClinicalTrials.gov.

Due to the rarity of the disease, there is little experience with treating several of these patients within one treatment center. Pooling clinical experience on symptomatic therapies is therefore crucial. Our objective was to evaluate the previous treatment approaches and responses to antiseizure medication (ASM), thereby providing evidence to guide symptomatic treatment decisions.

## 2 MATERIALS AND METHODS

We report the clinical course of a male individual since he was diagnosed at the age of 6 years until his transition from pediatric to adult care at age 18.

In addition, we contacted international collaborators via the “GeneMatcher”, “NETRE” and “ARCA‐Registry” platforms to identify additional genetically confirmed ST‐1 cases.^15^, ^16^, ^17^ Clinical data were collected using a standardized data sheet, focusing on seizure, especially MS management.

We obtained ethics approval for the retrospective analysis of patients with rare neurological disorders from the Leipzig University Hospital Ethics Committee (077/24‐ek). Patients gave their informed consent through their treating physicians and all data were processed pseudonymized.

Furthermore, a PubMed literature search was conducted for studies and case reports published between January 2010 and September 2025 using the terms “sialidosis” and “*NEU1*.” We included case reports that provided information about therapeutic interventions and placed particular emphasis on identifying reports involving treatment with ASM.

To assess treatment response in our cohort, we evaluated ASM response for BTCS and MS separately. ASM efficacy was classified as no response (−), mild improvement despite persistent seizures (+), marked improvement despite persistent seizures (++), or seizure freedom (+++). Temporary seizure freedom, noted in some cases, was additionally recorded as “temporarily” (Table 1). From the reviewed literature, ASM response could only be classified based on wording used. We incorporated these descriptions into Table S1 and categorized them per our scheme above.

**TABLE 1:** Demographic and diagnostic results from our cohort.

Case	1	2	3	4	5	6	7	8
Demographics								
Previously published	Yes	Yes	No	Yes	No	No	Yes	No
Previous publication	Assenza et al. (2021)^19^; Coppola et al. (2020)^21^; Rossi et al. (2020)^22^	Vélez Gómez et al. (2021)^20^		Mütze et al. (2016)^18^			Assenza et al. (2021)^19^; Coppola et al. (2020)^21^	
Gender	F	F	F	M	F	F	F	M
Age at last visit	32 years	55 years	17 years 3 months	17 years 11 months	18 years	24 years	23 years	66 years
Country of origin	Italy	Spain	Taiwan	Germany	Mexico	Turkey	Italy	USA
Genetic results								
Zygosity	Compound heterozygous	Homozygous	Compound heterozygous	Compound heterozygous	Compound heterozygous	Compound heterozygous	Compound heterozygous	Compound heterozygous
Variant 1	c.272 T>G	c.403G>A	c.544A>G	c.699C>A	c.893C>T	c.914G>A	c.982G>A	c.1084C>T
Variant 2	c.982G>A	c.403G>A	c.1021 + 1GA	c.803A>G	c.928G>A	c.1180G>A	c.1208delG	c.1247G>C
Other diagnostic results								
Metabolic results	Absent neuraminidase activity in fibroblasts	Low neuraminidase activity in fibroblasts	Low neuraminidase activity in fibroblasts	Low neuraminidase activity in fibroblast, normal in urine, abnormal oligosaccharide pattern in urine	ND	ND	ND	Normal results of oligosaccharides, free and total sialic acid
Brain‐MRI	Cerebellar atrophy (17 years and 23 years)	Normal (54 years)	Normal (13 years)	Normal (9 years and 15 years)	Normal (14 years)	Normal (19 years and 24 years)	Mild atrophy of superior part of vermis cerebelli (17 years)	Moderate cerebral and mild cerebellar volume loss, bifrontal; t2/flair hyperintensities: consistent with smaller vessel ischemic disease (65 years)
Ophthalmological findings	Cherry‐red macula spot + cataract	Cherry‐red macula spot	Cherry‐red macula spot + cataract	Cherry‐red macula spot + cataract	ND	ND	Cherry‐red macula spot + cataract	No
EEG	Interictal bilateral epileptiform discharges (13 years); diffuse polyspikes accompanied by intense myoclonus (27 years)	Normal (39 years)	Bilateral epileptiform disacharges (13 years)	Bilateral spike‐waves (15 years)	Bilateral polyspikes and spike‐waves	Normal	Bilateral slowing of background activity, focal epileptiform discharges (16 years)	Moderate bilateral slowing
SSEP	Giant potentials	Giant potentials	Giant potentials	Normal	ND	Normal	Normal	ND
VEP	Pathologic	ND	Pathologic (13 years)	Pathologic	ND	ND	ND	ND
EMG/ENG	Normal	ND	ND	Normal	Normal	Normal	Normal	Sensory neuropathy/neuropathy with preserved bilateral h‐reflexes

## 3 RESULTS

### 3.1 Case report

Our index case (published 2016 by Mütze et al.)^18^ was brought to our attention after a cherry‐red macular spot was noticed at age 6 years during a routine eye examination. Diagnosis of ST‐1 was confirmed by reduced neuraminidase activity in fibroblasts and detection of two variants of uncertain significance c.699C>A (p.Ser233Arg) and c.803A>G (p.Tyr268Cys) in *NEU1*, leading to amino acid changes predicted to impair protein function. The variant c.699C>A was maternally inherited; the father was not available for segregation analysis. Developmental milestones were reported as normal, and initial neurologic examination showed no abnormalities.

At age 12, he developed ataxia (SARA score: 5/40), hyperreflexia, saccadic eye movements, and horizontal nystagmus. Myoclonic seizures (MS) of the lower limbs emerged at age 14, triggered by sensory stimuli, leading to frequent falls and loss of ambulation. At age 15, he experienced a single bilateral tonic–clonic seizure (BTCS); interictal electroencephalography (EEG) recording showed paroxysms of irregular, bilateral synchronous spike‐waves.

Treatment with Levetiracetam (LEV) started at the age of 15, produced initial MS relief and restored ambulation, but the effect was transient. Dose escalations up to 2000 mg twice daily (≈80 mg/kg) yielded only short‐lived benefit with regard to MS. Perampanel (PER) add‐on from the age of 16 also suppressed MS transiently for a few months, requiring titration to the maximum licensed dose of 12 mg. By age 17, ataxia had progressed (SARA score: 11/40); also MS became more severe and he self‐up‐titrated LEV and PER to more than three times recommended doses (LEV 4000 mg/three times a day, PER 12 mg/three times a day), tolerated severe side effects (fatigue, drowsiness, dizziness, hallucinations, aggressions), yet ultimately lost independent ambulation. Due to treatment failure, valproate (VPA) was initiated with initial improvement on MS at doses of 30 mg/kg/d. Subsequent LEV taper worsened symptoms, necessitating maintenance at 50 mg/kg. Cognitive function remained intact. The patient relocated at age 18 and was lost to follow‐up.

### 3.2 Cohort analysis

Through international collaboration, we identified eight genetically confirmed ST‐1 patients (including the index case), summarized in Tables 1, 2, 3. Four of eight individuals were previously reported.^18^, ^19^, ^20^, ^21^, ^22^ However, we re‐evaluated the data at a later time point under ongoing treatment by collaborators and with a revised research focus.

Four individuals exhibited bilateral epileptiform discharges on EEG, one showed focal discharges, two had generalized slowing, and two had unremarkable findings. Cerebellar atrophy was observed on MRI in three individuals (two at age 17 years, one at age 65 years), cerebral volume loss at age 65 years in one individual. Cranial MRI in the remaining five patients was reported as normal (MRIs performed at ages between 9 and 54 years). Giant sensory evoked potentials were observed in 3 of 6 individuals. Visually evoked potentials were pathological due to latency delay in all 3 individuals examined. Electromyography and electroneurography were unremarkable in 5 of 6 individuals, but one individual aged over 60 years showed signs of sensory neuropathy (Table 1).

All individuals had age‐appropriate development until first symptoms of ST‐1. Symptom onset occurred in 6 of 8 individuals in adolescence and in 2 of 8 during middle adulthood.

The age at last visit ranged from 17 to 66 years (mean 31.6 years). Cognitive function remained intact in 5 of 8; two showed mild deficits; and one developed progressive decline after the age of 55 (Tables 1 and 2). Hyperreflexia was present in 5 of 8 individuals but only 1 of 8 showed spasticity. All developed ataxia, which was already classified as severe in some cases during adolescence. Six of eight individuals developed BTCS between the ages of 13 to 55 years (on average 23.5 years), which led to regression in only one case. MS occurred earlier between 12 and 48 years (mean 19.4 years), were reported in all individuals, and caused rapid loss of motor skills and walking ability. Alongside the decline in motor skills, 4 of 8 individuals developed psychiatric problems, including anxiety and depression (4 of 8) and, in two of these cases, also aggressive behavior (Table 2).

**TABLE 2:** Phenotypical and neurological profile in our cohort.

Case	1	2	3	4	5	6	7	8
Primary development	Normal	Normal	Normal	Normal	Normal	Normal	Normal	Normal
Initial cognitive skills	Short term memory deficit, otherwise normal	Mild intellectual disability	Normal	Normal	Normal	Normal	Normal	Normal
Dysmorphic features								
								Coarse facial features, medial eyebrows thicker than lateral
Regression								
Losing ability to walk	23 years	51 years	No	16 years	17 (gained ability back after starting PER)	26 years	18 years	66 years
Losing ability to write	20 years	NN	No	No	No	NN	18 years	
Dementia	No	No	No	No	No	No	No	Cognitive decline (since 55 years: problems with focused attention, word‐finding, worsening memory, making significant math calculation errors, difficulty with multitasking)
Psychiatric problems								
Depression/anxiety	Yes	No	No	Yes	Yes	No	No	Yes
Aggressive behavior	Yes	No	No	Yes	No	No	No	
Neurological profile								
Hyperreflexia	Yes	No	Yes	Yes	No	Yes	Yes	No
Spasticity	No	No	No	No	No	Severe (since 16 years)	No	No
Ataxia	Severe (since 17 years)	Severe (since 44 years)	Medium (since 14 years)	Medium (since 12 years)	Severe (since 15 years)	Medium (since 18 years)	Severe (since 16 years)	Severe + weakness of lower limbs (since 38 years)
Tremor	Severe (since 13 years)	No	No	Medium (since 13 years)	Severe (since 13 years)	Medium (since 16 years)	Medium	NN
Dystonia	No	No	No	No	No	Severe (since 25 years)	No	No
Dysarthria	Medium(since 23 years)	Medium (since 44 years)	Mild (since 15 years)	Mild (since 18 years)	Mild	No	Yes	Mild (since 66 years)
Dysphagia/oral feeding problems	No	With feeding problems (since 53 years)	With mild feeding problems (since 15 years)	No	No	No	No	Mild (since 66 years)
Neuropathy	No	No	No	No	No	No	No	Yes (diminished distal vibratory sensation)
Sensory sensations in limbs	NN	NN	Yes (since 14 years)	Yes (since 13 years)	NN	NN	NN	No
Myoclonic seizures (MS)	Severe (since 13 y)	Severe	Medium (since 13 years)	Medium (since 12 years)	Severe (since 15 years)	Severe (since 25 years)	Medium (since 12 years)	Mild and occasional (since 48 years, involving face, upper and lower extremities)
Epilepsy other than MS	Yes (since 25 years)	Yes (since 55 years)	Yes (since 13 years)	Yes(since 15 years)	Yes (since 14 years)	Yes (since 19 years)	No	No

In our cohort, a total of 14 antiseizure medications (ASM) were used, either as monotherapy or in combination (mean 5.4/patient, range 1–10; mean 3.3 concurrent at last follow‐up). Table 3 lists the frequency of ASM used in our cohort: ACZ (3), BRV (3), CBZ (1), CLB (1), CZP (5), GBP (1), LTG (1), LEV (7), OXC (1), PER (7), PIR (3), TPM (1), VPA (7), ZNS (2). At last visit, AZA (3), BRV (1), CZP (4), LEV (6), PER (6), VPA (6), ZNS (1).

**TABLE 3:** Occurrence of BTCS and MS and response to medication in our cohort.

Case	1	2	3	4	5	6	7	8
Epilepsy (other than MS)								
Age of onset	25 years	55 years	13 years	15 years	14 years	19 years	No	No
Seizure‐type	BTCS	BTCS	BTCS	BTCS	BTCS	BTCS	−	−
Regression coinciding with onset of epilepsy	No	No	No	No	Yes	No	−	−
Myoclonic seizures (MS)								
Age of onset	13 years	30 years	13 years	12 years	15 years	25 years	12 years	48 years
Motor regression coinciding with onset of myoclonus	Yes	Yes	Yes	Yes	Yes	Yes	Yes	No
Antiseizure medication								
Acetazolamid (ACZ)	Currently		Currently				Currently	
Response on seizures	+		+					
Response on myoclonus	+		+				+	
Brivaracetam (BRV)		Previously			Previously			Currently
Response on seizures		−			+			
Response on myoclonus		−			−			+
Carbamazepine (CBZ)	Previously							
Response on seizures	−							
Response on myoclonus	−							
Clobazam (CLB)		Not tolerated						
Response on seizures		?						
Response on myoclonus		?						
Clonazepam (CZP)	Currently	Currently	Previously				Currently	Currently
Response on seizures	+	−	−					
Response on myoclonus	++	+	+				+	+
Gabapentin (GBP)		Previously						
Response on seizures		−						
Response on myoclonus		−						
Lamotrigine (LTG)					Previously			
Response on seizures					+			
Response on myoclonus					−			
Levetiracetam (LEV)	Currently	Currently	Currently	Currently	Currently		Currently	Previously
Response on seizures	+	−	+	+++	+			
Response on myoclonus	+	+	+	++ temporarily for a few weeks, then dosage increase required	−		+	−
Oxcarbazepine (OXC)					Previously			
Response on seizures					+			
Response on myoclonus					−			
Perampanel (PER)	Currently	Previously	Currently	Currently	Currently		Currently	Previously
Response on seizures	++	−	+	+++	+			
Response on myoclonus	+++	−	+	++ temporarily for a few weeks, then dosage increase required, patient overdoses to achieve better effect despite side effects	+++ temporarily for a few weeks, then dosage increase required, the response is not constant she has good and bad days, but regain some authonomy back		+++	Not tolerated
Piracetam (PIR)	Previously	Previously	Previously					
Response on seizures	−	−	−					
Response on myoclonus	+	−	−					
Topiramate (TPM)		Previously						
Response on seizures		−						
Response on myoclonus		−						
Valproate (VPA)	Currently	Previously	Currently	Currently	Currently	Currently		Currently
Response on seizures	+	−	+	+++	+	+++		
Response on myoclonus	−	−	+	+ temporarily for a few weeks, then dosage increase required	−	+++		+
Zonisamide (ZNS)	Previously	Currently						
Response on seizures	−	+						
Response on myoclonus	+	+						
Other medication								
	Escitalopram					Propranolol		Hydroxyzine Amiodarone Metoprolol

The ASM reported as effective on BTCS were: ACZ 2/2; BRV 1/2; CBZ 0/1; CZP 1/3; GBP 0/1; LTG 1/1; LEV 4/5; OXC 1/1; PER 4/5; TPM 0/1; VPA 5/6; ZNS 1/2.

The ASM reported as effective on MS were: ACZ 3/3; BRV 1/3; CBZ 0/1; CZP 5/5; GBP 0/1; LTG 0/1; LEV 5/7; OXC 0/1; PER 5/7; TPM 0/1; VPA 4/7; ZNS 2/2.

ACZ, LEV, and VPA were the most effective against BTCS; ACZ, CZP, LEV, PER, VPA, and ZNS against MS. Improvement of MS was observed in all patients treated with ACZ, CZP, and ZNS. LEV and PER often yielded substantial yet sometimes transient benefit, occasionally requiring high dosing.

### 3.3 Literature review

We performed a PubMed literature search (January 2010–September 2025) for ST‐1 publications. Given the scarcity of published cases, we sought to include all available reports on therapeutic approaches. Case reports without individualized medication details per patient were excluded. Heterogeneous reporting styles complicated treatment response categorization. No studies provided quantitative metrics, requiring descriptive classification per our cohort scheme. Distinguishing “mild” from “marked” improvement (as in our cohort) was impossible based on wording or polytherapy use, so we merged them into “partially effective.” Thus, we condensed the original four categories into three for overall assessment. Final categories were: “not effective,” “partially effective,” and “effective.”

In 20 publications, we obtained information on therapeutic approaches for epilepsy, particularly for MS, covering a total of 27 reported cases. 21, 23, 24, 25, 26, 27, 28, 29, 30, 31, 32, 33, 34, 35, 36, 37, 38, 39, 40, 41 A tabular summary can be found in Table S1. Summarized analysis of our cohort of 8 patients and 25 previously published cases can be found in Table 4.

**TABLE 4:** The overall therapeutic response to antiseizure medication (ASM) in our cohort is presented below. The total number of published cases (from January 2010 to September 2025) is provided in parentheses. Patients whose therapeutic response could not be reliably assessed (e.g., due to poor medication tolerability, insufficient clinical information, or because the ASM was part of a polytherapy regimen) are listed in the column “uncertain effect.” To avoid double counting, two individuals from the publication by Coppola et al. (2020) were included in our cohort but not in the count of published cases.

ASM	Number of patients	Effect on BTCS			Effect on MS			Temporarily effective	Uncertain effect
		Not effective	Partially effective	Effective	Not effective	Partially effective	Effective		
AZA	3 (0)		2/2			3/3			
BRV	3 (0)		1/2		2/3	1/3			
CBZ	1 (1)	1/1			1/1 (1/1)				
CLB	1 (3)				(2/3)				1 (1)
CZP	5 (14)	2/3 (2/6)	1/3		(4/14)	4/5 (4/14)	1/5		(6)
GBP	1 (0)	1/1			1/1				
LTG	1 (0)		1/1		1/1				
LEV	7 (17)	1/5	3/5 (4/12)	1/5 (1/12)	2/7 (4/17)	4/7 (5/16)	1/7 (1/16)	1/7 (2/16)	(7)
OXC	1 (0)		1/1		1/1				
PB	1							1/1	1/1
PER	7 (5)	1/5 (1/3)	2/5	2/5	1/7	1/7 (3/5)	4/7 (1/5)	2/7	1/7 (1)
PIR	3 (1)	3/3 (1/1)			2/3 (1/1)	1/3			
PGM	(1)								(1)
PRM	(1)	(1/1)			(1/1)				
TPM	1 (4)	1/1			1/1		(1/4)	(3/4)	(1)
VPA	7 (16)	1/6 (1/7)	3/6	2/6	3/7 (5/16)	3/7 (3/16)	1/7 (1/16)	(6/16)	(2)
ZNS	2 (3)	1/2	1/2		(1/3)	2/2		(2/3)	
Deep brain stimulation	(1)						(1/1)		
Ketogenic diet	(1)				(1/1)				
Sodium oxybate	(2)					(1/2)	(1/2)		

We excluded two cases from the literature review because they were identical to cases already included in our cohort compilation.^21^

All included cases (25 of 25) presented with MS, and 13 of 25 also had BTCS. The most commonly used ASMs overall were LEV (*n* = 24), VPA (*n* = 23), and CZP (*n* = 19). In recent years, PER has been used increasingly (*N* = 12).

In the pooled analysis of 33 cases, ACZ led to a positive response for both BTCS and MS in all three treated patients. PER was effective against BTCS in two‐thirds of cases and against MS in 9 of 10 cases, although effects were only transient in two. VPA was effective for BTCS in 2 of 7 patients and improved MS in two‐thirds of patients, but its effect was transient in three individuals. LEV was effective in 9 of 10 cases for BTCS and in two‐thirds for MS, with transient responses in three patients. CZP was less effective against BTCS but improved MS in two‐thirds of patients.

In addition to antiseizure medication, several alternative therapeutic approaches were reported. One published case showed a very good response to deep‐brain stimulation.^27^ Another publication reported the use of sodium oxybate in two patients, both of whom responded well, although one did not tolerate the treatment.^30^ A ketogenic diet was implemented in one individual but proven ineffective.^36^

Preclinical fibroblast studies suggest that betaine supplementation or histone deacetylase (HDAC) inhibition with romidepsin can enhance residual NEU1 activity and metabolic function.^14^ However, no clinical evidence currently supports these findings.

## 4 DISCUSSION

ST‐1 is one of the various rare causes that lead to progressive myoclonus epilepsy (PME), a rare generalized epilepsy syndrome. Typical features include progressive MS, BTCS, and ataxia. Regardless of the underlying disease, the sudden, brief, shock‐like contractions in MS result in substantial patient disability and are difficult to treat. The exact mechanisms or underlying brain networks responsible for this specific phenotype remain poorly understood. Studies have identified white‐matter tract abnormalities, particularly in the cerebello‐thalamo‐cortical tract but also in other brain regions.^42^ Microglial activation may potentially play a role here, subsequently leading to neuroinflammation and neuronal loss.^43^, ^44^, ^45^

PER, LEV, VPA, ACZ, CZP, and ZNS demonstrate superior efficacy over other ASMs in PME other ST‐1, likely due to their distinct mechanisms of action. Specifically, PER selectively antagonizes AMPA receptors to disrupt cortico‐subcortical synchronization and cortical hyperexcitability;^19^ LEV binds synaptic vesicle 2A (SV2A) to modulate neurotransmitter release and attenuate hypersynchrony;^46^ VPA augments GABAergic inhibition while blocking voltage‐gated sodium/T‐type calcium channels and curbing glutamate release to target thalamo‐cortical networks;^47^ ACZ inhibits carbonic anhydrase, inducing intracellular acidification that enhances inhibitory chloride gradients and elevates seizure threshold;^48^ CZP allosterically potentiates GABA_A receptors for rapid suppression of myoclonus‐generating cortical hyperexcitability;^49^ and ZNS blocks sodium/T‐type calcium channels, bolsters GABAergic tone, and dampens glutamate to stabilize thalamo‐cortical circuits.^50^

Therapy for ST‐1 follows general therapeutic principles. However, the evidence base for ST‐1 therapy remains limited to case reports and small case series, with no randomized controlled trials available.

Based on our study, we recommend prioritizing PER, LEV, VPA, ACZ, CZP, and ZNS according to predominant seizure types (BTCS and/or MS).

Polytherapy—often involving three or more concurrent ASMs—increases side effect risk.

No ST‐1 cases treated with ethosuximide have been reported; it may warrant consideration for MS.

No evidence suggests ASMs exacerbate myoclonus in ST‐1; however, CBZ, GBP, LTG, PHT, and VGB can worsen myoclonic epilepsy and should be avoided or used with caution.^26^, ^38^, ^51^

Despite initially good response to some ASMs for MS, the therapeutic effect waned over time in some patients. Whether treatment interruption upon waning efficacy, followed by later resumption, is advisable remains unresolved. However, ASMs address symptoms, not the underlying lysosomal defect. Given ST‐1's progressive nature—characterized by persistent substrate accumulation and resultant neuronal hyperexcitability—the ASM effect is likely transient.

Evidence for therapeutic approaches other than ASMs remains far weaker:

Sodium oxybate enhances inhibitory neurotransmission and treats cataplexy and excessive daytime sleepiness in narcolepsy. Off‐label, it has shown promise in single cases of refractory familial myoclonus‐dystonia,^52^ posthypoxic myoclonus,^53^ and two individuals with ST‐1.^30^ Nevertheless, like deep brain stimulation in ST‐1 (reported in one case only),^27^ it warrants further evaluation.

Additionally, clinical data in humans are still needed before evidence‐based recommendations can be made for dietary interventions—vitamin supplementation, HDAC inhibitors, or ketogenic/Atkins diets. The same applies to anti‐inflammatory therapy.

Future hope rests with enzyme replacement therapy or gene therapy.^11^, ^12^, ^13^, ^14^, ^44^ However no active phase I/II trials are registered on ClinicalTrials.gov yet.

A major limitation of our study is that, despite international collaboration, we could only include a small number of patients. This fundamentally poses a major challenge for very rare diseases. Additionally, the ST‐1 literature is limited by its primary focus on diagnostic clinical presentations, with scarce data on long‐term therapeutic outcomes. Medication effects in case reports were often not reported or described imprecisely. The evaluation of treatment efficacy is further complicated by the frequent use of polytherapy in published cases. This complicates the retrospective assessment of therapeutic effects.

Consequently, therapeutic recommendations remain provisional due to the small cohort size, heterogeneous treatment regimens, and limited follow‐up.

Given the rarity of ST‐1 and the need to improve patient care, systematic documentation of the natural disease course, therapeutic interventions, treatment responses, and tolerability of symptomatic therapies is essential. For this reason, an international registry would be highly valuable to collate data from as many patients as possible—notably on treatment response—allowing treating physicians and patients to contribute information.

In addition, rare disease centers, specialized epilepsy centers, and patient advocacy groups could coordinate longitudinal data collection and facilitate clinical trials.

## 5 CONCLUSION

ACZ, PER, LEV, VPA, CZP, and ZNS have demonstrated fairly consistent efficacy against BTCS and MS. However, the data available to date are very limited for providing clear treatment recommendations for sialidosis.

Given the absence of disease‐modifying therapies and substantial patient morbidity, systematic data collection via a patient registry is urgently needed to guide future symptomatic and curative trials.

## AUTHOR CONTRIBUTIONS

Conceptualization: Janina Gburek‐Augustat, I‐Chun Lee, Antonietta Coppola. Data collection: Janina Gburek‐Augustat, Antonietta Coppola, I‐Chun Lee, Marica Rubino, Vehap Topçu, Melissa Chavez‐Castillo, Shao Ching Tu, Marwan Shinawi, Isabel Alfradique‐Dunham, Manouela Valtcheva, Astrid Adarmes‐Gómez, Daniel Macias‐Garcia, Laura Muñoz‐Delgado, Silvia Jesús, Pablo Mir, Andreas Merkenschlager. Data analysis: Janina Gburek‐Augustat, Antonietta Coppola. Writing Original Draft: Janina Gburek‐Augustat. Writing‐Review and Editing: Antonietta Coppola, Marwan Shinawi, I‐Chun Lee, Marica Rubino, Vehap Topçu, Melissa Chavez‐Castillo, Shao Ching Tu, Isabel Alfradique‐Dunham, Manouela Valtcheva, Astrid Adarmes‐Gómez, Daniel Macias‐Garcia, Laura Muñoz‐Delgado, Silvia Jesús, Pablo Mir, Andreas Merkenschlager.

## FUNDING INFORMATION

There was no financial support for this study.

## CONFLICT OF INTEREST STATEMENT

The authors declare no competing interests related to this study.

## ETHICS STATEMENT

We confirm that we have read the Journal's position on issues involved in ethical publication and affirm that this report is consistent with those guidelines.

## Supporting information


Data S1:

## Data Availability

The data that support the findings of this study are available from the corresponding author upon reasonable request.
