# Supplementary material for: Sialidosis type I: How to alleviate disabling myoclonic seizures?—A multicenter analysis of eight cases and review of the literature
Source: Epilepsia Open. 2026 Feb 10;11(2):577–91. doi: 10.1002/epi4.70233 (PMC13052255; doi:10.1002/epi4.70233)
Supplement: Supplementary file 1 — Data S1: [file EPI4-11-577-s001.docx]

**Supplement Table 1:** Overview of published cases between January 2010 and September 2025 and their response to ASM

| Case | Male/Female  age at report  (age at onset) | variants in *NEU1* | presenting seizure type | ASM or other therapy | effect on BTCS | effect on MS | source |
| --- | --- | --- | --- | --- | --- | --- | --- |
| 1 | M  33y  (16y) | c.544A>G (homozygous) | BTCS (once long before ASM started)  MS | VPA +LEV |  | no | Zhou et al. (2025) |
|  |  |  |  | PER +VPA +LEV |  | yes |  |
| 2 | F  11y  (9y) | c.239C>T + c.880C>T (compound) | BTCS  MS | LEV | yes | yes | Ding et al. (2024) |
| 3 | F  (13y) | c.544A>G + c.428T>A  (compound) | BTCS  MS | VPA +CZP +TPM +LEV | reduced seizure frequency | reduced myoclonus | Li et al. (2024) |
| 4 | M  (14y) | c.544A>G + c.428T>A  (compound) | BTCS  MS | VPA +CZP +TPM +LEV | reduced frequency | reduced frequency | Li et al. (2024) |
| 5 | F  40y  (30y) | c.544A (homozygous) | MS | VPA +Fluphenazine +Bezocaine +Vitamin B1/B6/B12 |  | reduced frequency | Zhang et al. (2024) |
|  |  |  |  | Diazepam+  Agomelatine+  VPA | died in status epilepticus 10 days after establishing the previous combination therapy |  |  |
| 6 | M  19y  (14y) | c.239C>T + c.803A>G | MS | Baclofen +CZP +VPA +LEV |  | slightly relieved, gradually losed effectiveness | Liu et al. (2023) |
|  |  |  |  | DBS: bilaterale GPi electrodes |  | significant improvement |  |
| 7 | F  25y  (12y) | c.544A>G  (homozygous) | BTCS  MS | LEV | good on seizures | no effect | Wang et al. (2022) |
|  |  |  |  | VPA + CZP | NN | no effect |  |
| 8 | M  33y  (19y) | c.880C>T  (homozygous) | MS | CZP +VPA +LEV |  | mild improvement | Neeraja et al. (2021) |
| 9 | F  19  (7y) | c.544A>C + c.727G>A (compound) | MS | PGM burning sensation in hand and feet resolved |  | NN | Riboldi et al. (2021) |
|  |  |  |  | Sertraline (depression) |  | NN |  |
|  |  |  |  | Trihexyphenidyl |  | no |  |
|  |  |  |  | CZP |  | no |  |
|  |  |  |  | LEV |  | NN |  |
|  |  |  |  | alcohol |  | responsive |  |
| 10 | M  40y  (8y) | c.629C>T (homozygous) | MS | VPA |  | NN | Riboldi et al. (2021) |
|  |  |  |  | LEV |  | not tolerated |  |
|  |  |  |  | PER |  | not tolerated |  |
|  |  |  |  | CZP |  | NN |  |
|  |  |  |  | ZNS |  | NN |  |
|  |  |  |  | alcohol |  | responsive |  |
|  |  |  |  | sodium oxybate |  | improvement  (but not tolerated) |  |
| 11 | M  31y  (<12) | c.644T>C +  c.649G>A  (compound) | BTCS  MS | CZP |  | improvement | Riboldi et al. (2021) |
|  |  |  |  | +LEV | yes | improvement |  |
|  |  |  |  | alcohol |  | responsive |  |
|  |  |  |  | sodium oxybate |  | great improvement |  |
| 12 | M  22y  (15y) | c.239C>T + c.544A>G  (compound) | MS | Buspirone + Idebenone |  | discontinued | Cao et al. (2021) |
| 13 | M  15y  (13y) | c.872T>C (homozygous) | BTCS (just once)  MS | LEV |  | significant improvement | Mukherjee et al. (2021) |
|  |  |  |  | VPA |  | significant improvement |  |
| 14 | M  17y  (10y) | c.544A>G + c.1118T>C (compound) | BTCS  MS | LEV | yes | partial effect | So et al. (2020) |
|  |  |  |  | +PER |  | improvement |  |
| 15 | F  43y 13y) | c.982G>A + c.1208delG | BTCS  MS | PB / VPA / CZP / CLB / LEV / TPM / ZNS |  | mild and  transient effect | Coppola et al. (2020) |
|  |  |  |  | PER +VPA +CZP +LEV | reduced seizure frequency | improved |  |
| *16* | *F*  *28y*  *(13y)* | *c.272T>F + c.982G>A (compound)* | *BTCS*  *MS* | *VPA +CZP +LEV +PIR +AZA +ZNS* |  |  | *Coppola et al. (2020)*  *is the same as case 1 in our cohort* |
|  |  |  |  | *PER +CZP +AZA +LEV* |  | *disappearance of MS* |  |
| 17 | F  41  (17y) | c.914G>A + c.625delG (compound) | BTCS  MS | VPA +CZP +PRM +PIR | ongoing, then ceased at 38y | ongoing | Coppola et al. (2020) |
| *18* | *F*  *18y*  *(12y)* | *c.982G>A + c.1208delG (compound)* | *MS* | *LEV +CZP +AZA* |  | *short-term benefit* | *Coppola et al. (2020)*  *Is the same as case 7 in our cohort* |
|  |  |  |  | PER |  | *siginificant benefit* |  |
| 19 | M  17y  (childhood) | c.239C>T + c.803A>G (compound) | BTCS  MS | LEV | good response | started under LEV | Lv et al. (2020) |
| 20 | M  16y  (5y) | c.314_352del+ c.544A>G | MS | LEV |  | good response | Fan et al. (2019) |
| 21 | M  15y  (12y) | c.544A>G + c.619C>T (compound) | MS | VPA / LEV / CLB / PB / ketogenic diet |  | ineffective | Hu et al. (2018) |
|  |  |  |  | PER |  | remission |  |
| 22 | M  24y  (18y) | c.625delG + c.928G>A  (compound) | BTCS  MS | LEV | response | response | Gultekin et al. (2018) |
| 23 | M  39y  (16y) | c.629C>T  (homozygous) | BTCS  MS | VPA / ZNS / CZP |  | poor effect | Aravindhan et al. (2018) |
| 24 | M  17y  (14y) | c.239C>T + c.403G>A | MS | CBZ |  | no effect | Sekijima et al. (2013) |
|  |  |  |  | CZP |  | improvement |  |
| 25 | F  16y  (11y) | NN | MS | VPA + PRM+ CLB |  | ongoing MS and development of a myoclonic SE | Bragatti et al. (2011) |
|  |  |  |  | TPM |  | discontinuation of myoclonic SE and improvment while continuing |  |
| 26 | M  29y  (14y) | c.544A>G (homozygous) | MS | CZP +VPA |  | moderate improvement | Chen et al. (2006) |
| 27 | F  31y  (17y) | c.544A>G (homozygous) | MS | CZP +VPA |  | moderate improvement | Chen et al. (2006) |
